# SECT-Net: hybrid dual-decoder network with SE-convolution transformer for liver tumor segmentation

**DOI:** 10.3389/fphys.2026.1753152

**Published:** 2026-03-11

**Authors:** Qiquan Zeng, Dongfen Ye, Meiqin Chen, Xiaoliang Jiang

**Affiliations:** 1 College of Mechanical Engineering, Quzhou University, Quzhou, China; 2 College of Electrical and Information Engineering, Quzhou University, Quzhou, China; 3 Department of Infectious Diseases, The Quzhou Affiliated Hospital of Wenzhou Medical University, Quzhou People’s Hospital, Quzhou, China

**Keywords:** deep feature capture module, dual-decoder network, encoder-decoder, liver tumor segmentation, SE-convolutionTransformer module

## Abstract

**Introduction:**

Early accurate diagnosis of liver tumors plays a pivotal role in improving patient prognosis and guiding effective treatment planning. However, the automated segmentation of liver tumors remains a highly challenging task due to several intrinsic factors, including heterogeneous intensity distribution, blurred or indistinct boundaries, irregular tumor shapes, and wide variations in size and appearance across patients. To overcome these limitations, we propose a hybrid dual-decoder network that integrates squeeze-and-excitation convolution (SE-convolution) and Transformer-based attention mechanism for liver tumor segmentation.

**Methods:**

Specifically, SECT-Net adopts the classical encoder-decoder architecture as its foundation and introduces a dual-decoder mask mechanism to enhance feature discrimination during segmentation. To enhance the encoder’s capability in capturing both global contextual dependencies and fine-grained local features, the SE-convolution Transformer module (SECTM) is integrated into the second, third, and fourth layers of the encoder. Furthermore, a deep feature capture module (DFCM) is embedded at the bottleneck layer to enhance the network’s ability to extract and preserve high-level semantic representations. After that, the extracted deep features are seamlessly integrated through skip connections with the decoder.

**Results and Discussion:**

To comprehensively assess the effectiveness and generalization capability of SECT-Net, extensive experiments were conducted on the liver tumor datasets collected from Quzhou People’s Hospital. On the arterial phase dataset, SECT-Net demonstrated excellent segmentation performance, achieving Dice of 0.8452, Mcc of 0.8411, and Jaccard of 0.7339. Similarly, on the portal venous phase dataset, SECT-Net maintained robust generalization, with Dice of 0.8425, Mcc of 0.8396, and Jaccard of 0.7339. Furthermore, on the public 3DIRCADb dataset, SECT-Net also achieved competitive performance, with Dice, Mcc, and Jaccard scores of 0.8845, 0.8855 and 0.7969. These consistent results across both private and public datasets further demonstrate the strong reliability, robustness, and generalization capability of SECT-Net in segmenting liver tumors with diverse intensity distributions and morphological characteristics.

## 1 Introduction

With the rapid advancement of modern society and the continuous acceleration of industrialization, the incidence and mortality rates of malignant tumors have been steadily increasing worldwide. Among these malignancies, liver cancer stands out as one of the most prevalent and life-threatening diseases, posing a major global health challenge. Early diagnosis and precise treatment of liver tumors are crucial for improving patient survival and prognosis. However, the considerable variations in tumor morphology, size, and anatomical location among different patients present significant challenges for accurate clinical diagnosis and treatment planning. In recent years, radiotherapy, local ablation, and targeted therapy have become the main therapeutic strategies for liver cancer, all of which rely heavily on medical imaging technologies. Among various modalities, computed tomography (CT) has emerged as the preferred imaging technique due to its high efficiency, accessibility, and cost-effectiveness. Nevertheless, liver tumor segmentation in CT images is still largely performed manually by radiologists. This work is not only time-consuming and labor-intensive, but also prone to unstable segmentation results due to human factors. Therefore, developing an automatic liver tumor segmentation method has become a crucial research direction, as it can provide valuable auxiliary support for disease diagnosis, preoperative planning, and treatment monitoring.

In recent years, deep learning has emerged as a transformative technology in medical image analysis and has been extensively applied to the segmentation of liver tumors in CT images. Benefiting from their strong hierarchical feature learning and representation abilities, convolutional neural networks (CNNs) have achieved remarkable success in accurately identifying complex anatomical structures and delineating tumor boundaries. Through multiple nonlinear transformations, CNNs can automatically capture rich spatial textures, intensity variations, and multi-scale contextual dependencies, effectively reducing the reliance on handcrafted features. With the continuous advancement of network architectures, numerous hybrid deep learning models have been developed that combine attention mechanisms (Wu et al., 2026; Mei and Yu, 2025), Transformer components (Raghaw et al., 2025; Sivanagaraju et al., 2025), and multi-scale strategies (Hu Y et al., 2025; Li et al., 2023) to achieve more accurate and robust liver tumor segmentation. For example, Zhu et al. (2026) introduced a biomimetic parallel path network designed to simulate the cooperative information-processing mechanisms observed in biological visual systems. By constructing multiple parallel feature extraction pathways, the network can effectively integrate both global context and local detail information. Ma et al. (2026) developed a multi-scale spatial-frequency feature enhancement framework that combines spatial and frequency information to improve feature learning. Jin et al. (2025) designed a longitudinal collaborative segmentation mechanism aimed at modeling temporal dependencies among images acquired at different time points. Yang et al. (2025) introduced an enhanced segmented multi-layer perceptron module aimed at optimizing feature dimensionality reduction while preserving critical spatial and semantic information. Zhao and Li (Zhao and Li, 2025) developed an uncertainty-guided module designed to adaptively refine feature learning by quantifying prediction confidence during the segmentation process. Cheng et al. (2024) developed an edge-guided attention refinement method that focuses on enhancing the accuracy of tumor boundary delineation. Prabaharan et al. (2024) proposed an artificial intelligence-based framework that integrates a histological convolutional autoencoder to enhance feature representation and learning efficiency.

Although deep learning-based methods have achieved impressive advancements in liver tumor segmentation, there are still some major challenges in clinical practice. Firstly, there are significant differences among patients, as tumors vary in size, shape, boundary definition, and spatial distribution. Secondly, medical images typically contain abundant anatomical details along with noise and artifacts, while the contrast between tumors and surrounding tissues is often low, especially in early-stage lesions or specific pathological states. Additionally, differences introduced by different imaging devices, acquisition settings, or reconstruction algorithms further challenge the model’s robustness and consistency. Finally, the limited availability of high-quality annotated datasets and the subjectivity of expert labeling pose additional constraints on model training and generalization.

Considering the aforementioned challenges, this study introduces a novel segmentation framework termed SECT-Net, specifically designed to address the complexity of liver tumor segmentation. The proposed SECT-Net architecture integrates two core functional modules: the SE-convolution Transformer module and the deep feature capture module. The former leverages the SE module and Transformer mechanism to effectively capture global contextual dependencies while preserving fine-grained local details, while the later focuses on enhancing the extraction and retention of high-level semantic representations. Notably, these modules operate collaboratively within an encoder-decoder framework equipped with a dual-decoder mask mechanism, ensuring the stability and efficiency of feature interaction. The contributions of this work are summarized as follows.

1. We propose a hybrid dual-decoder segmentation network equipped with an explicit decoder-mask interaction mechanism. In this framework, the first decoder produces a coarse segmentation mask, which is subsequently injected into the feature learning process of the second decoder to guide feature refinement. This mask-guided cross-decoder interaction enables coarse localization cues to be effectively transformed into fine-grained, boundary-aware representations, leading to improved structural delineation.
2. We introduce a SE-convolution Transformer module into the second, third, and fourth encoder stages to enhance hierarchical feature representation. The core innovation of this module lies in the design of a bilinear attention mechanism, which explicitly models second-order interactions between spatial features. By integrating bilinear attention with SE-ASPP-based multi-scale context modeling and convolutional refinement, the SECTM effectively strengthens global contextual perception while preserving fine-grained local structures.
3. At the bottleneck stage, we design a deep feature capture module to explicitly enhance high-level semantic aggregation before decoding. By coupling attention-guided feature refinement with cross-level feature concatenation and skip connections, the DFCM facilitates more coherent semantic fusion and provides a structurally enhanced feature representation for subsequent decoder reconstruction, leading to improved boundary sharpness and segmentation consistency.

## 2 Related work

### 2.1 Encoder-decoder framework

The encoder-decoder architecture has emerged as a fundamental paradigm in medical image segmentation because of its capacity to encode complex visual patterns and reconstruct fine spatial details with high precision. Within this framework, the encoder progressively transforms input images into compact, semantically rich representations, while the decoder restores spatial information through hierarchical up-sampling and feature aggregation. Owing to these strengths, U-Net (Ronneberger et al., 2015) and its numerous improved derivatives have become benchmark models, consistently achieving outstanding segmentation results across a wide range of medical imaging applications. For instance, Hu Z et al. (2025) enhanced the traditional encoder-decoder segmentation architecture by embedding an edge feature enhancement module within the codec framework. This module is specifically designed to strengthen the representation of boundary-related information, which enables the network to better preserve edge continuity. Wang et al. (2025a) proposed a multi-semantic progressive interaction guidance mechanism designed to enhance feature communication across different semantic levels within the network. Bansal and Vidyarthi (2025) proposed a hierarchical meta-heuristic encoder-decoder architecture that integrates multi-level feature extraction with adaptive optimization strategies to improve segmentation performance. In this framework, the encoder hierarchically captures spatial and semantic representations across different abstraction levels, while the decoder employs meta-heuristic principles to dynamically refine and reconstruct feature maps. Xie A et al. (2025) enhanced the segmentation framework by integrating a boundary and shape guidance strategy aimed at improving the accuracy of contour delineation and structural consistency. Dhinakaran et al. (2025) introduced a distributed multi-kernel classification framework designed to efficiently model data complexity while preserving the most informative features of the input images. Kalpana et al. (2025) proposed a context-aware spatial decomposition network aimed at mitigating the adverse effects of noise and improving feature robustness.

### 2.2 Transformer-based mechanism

In recent years, the Transformer architecture has gained widespread recognition in computer vision and medical image segmentation owing to its exceptional capacity to capture long-range feature dependencies and model global contextual interactions. Unlike traditional convolutional networks constrained by limited receptive fields, Transformers utilize a self-attention mechanism that dynamically measures the relationships among all feature positions within an image. Consequently, Transformer-based approaches have achieved significant breakthroughs in segmentation tasks that demand precise boundary identification, structural consistency, and comprehensive contextual understanding. For example, Zhou et al. (2026) developed a frequency cross-attention block built upon the Transformer framework to strengthen global-local feature interactions in image representation learning. Qiu et al. (2026) enhanced the traditional vision Transformer framework by integrating spatial dynamic components that enable the network to adaptively adjust its attention distribution according to spatial context. Mi and Zhang (2025) introduced a distance-aware Transformer that enhances the conventional self-attention mechanism by incorporating spatial distance information between image tokens. Pang et al. (2025) introduced a knowledge-guided polar coordinate Transformer, which incorporates prior geometric knowledge and spatial priors into the Transformer framework to improve feature representation and contextual reasoning. Wang T. et al. (2025) proposed a Transformer architecture with multi-branch enhanced memory designed to improve the model’s ability to retain and utilize contextual information across different feature hierarchies.

### 2.3 Dual-path structure

The dual-path architecture has emerged as an effective paradigm in image segmentation for its capacity to extract and integrate complementary feature representations in parallel. Compared with single-path networks, dual-path models achieve a more balanced trade-off between semantic abstraction and structural precision. For instance, Xiao et al. (2025) proposed a dual-path feature fusion module that performs feature extraction through two collaborative branches, thereby facilitating more efficient interaction between heterogeneous feature representations. Specifically, the convolutional branch is designed to capture local spatial textures and fine structural details, while the state-space branch focuses on modeling long-range dependencies and contextual correlations. Xie Q et al. (2025) employed a dual-path spatial feature encoding strategy to extract complementary information from both the foreground regions and object edges. Dai et al. (2024) leveraged the rich bidirectional information exchange between two parallel feature extraction paths to enhance the overall representational capacity of the network. Fang and Jiang (2024) developed a dual-pathway module that analyzes and integrates diverse feature representations corresponding to different intracranial hematoma subtypes. Jiang et al. (2023) designed a dual-path complementary mechanism aimed at effectively integrating multi-level features derived from both the multilayer perceptron and convolutional neural network. Yang and Li (2023) introduced a dual-path Transformer architecture designed to capture complementary representations by modeling global dependencies and local contextual information in parallel.

## 3 Methods

In this section, we give a clear overview of the SECT-Net architecture, outlining its design motivations and primary workflow. Next, we provide a detailed description of its two key components: SE-convolution Transformer module and deep feature capture module. Finally, we introduce the loss function used in SECT-Net.

### 3.1 Overview architecture of SECT-Net

**FIGURE 1:**
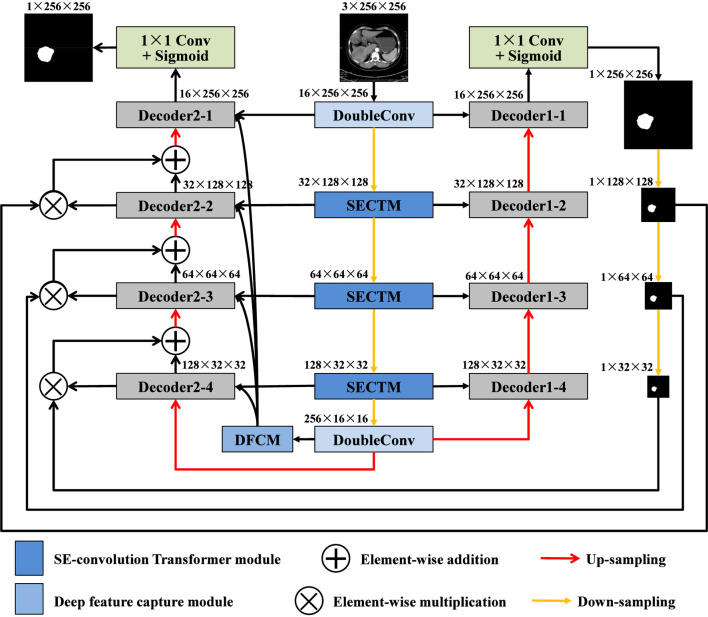
Overview architecture of SECT-Net.

In most conventional designs, dual decoders operate either independently or are loosely fused through feature concatenation or summation, which may still suffer from redundant feature propagation and insufficient suppression of background noise. To overcome this issue, we propose a new architecture named SECT-Net, which enhances feature selection during decoding by introducing a dual-decoder mask mechanism. As illustrated in Figure 1, SECT-Net follows a standard encoder and decoder structure while incorporating two collaborative decoders that interact in a guided manner. Specifically, the encoder begins with a DoubleConv block (two 3 × 3 convolutions) that extracts the initial low-level features from the input image. It is followed by down-sampling operations and three SE-convolution Transformer modules that progressively capture multi-scale contextual information. The deepest encoder layer is further refined by an additional DoubleConv block to strengthen high-level semantic representations. On the right side of the network, Decoder1 adopts the traditional U-Net decoding strategy. Features from the encoder are concatenated with the up-sampled features in Decoder1 and processed by DoubleConv blocks. A final 1 × 1 convolution with a sigmoid activation generates a coarse mask that highlights potential tumor regions. On the left side of the network, Decoder2 incorporates this mask guidance during its decoding process. The final encoder features are first processed by the deep feature capture module, and the resulting enhanced features are distributed to each stage of Decoder2. Meanwhile, the mask generated by Decoder1 is resized to match the spatial resolution of each decoding layer in Decoder2. These resized masks are then fused with the corresponding Decoder2 features through element-wise multiplication, which strengthens tumor-related regions and suppresses background noise. Through this collaborative mechanism, Decoder1 provides region-level guidance, and Decoder2 performs refined feature reconstruction based on the mask information. This design enables SECT-Net to effectively filter out irrelevant information, focus on important structures, and achieve more accurate and robust liver tumor segmentation. Compared with existing dual-path or dual-decoder segmentation frameworks, which usually operate in a parallel or loosely coupled manner, SECT-Net introduces an explicitly guided interaction mechanism between the two decoders. Instead of independently refining features, Decoder1 first generates a coarse but semantically meaningful mask that provides region-level prior knowledge. This mask is then used to guide Decoder2 at multiple scales through element-wise modulation, selectively enhancing tumor-related responses while suppressing background noise. Furthermore, by combining the deep feature capture module with attention-based refinement, SECT-Net enables more selective and semantically aligned feature transmission, effectively reducing noise propagation and semantic inconsistency. As a result, SECT-Net achieves more accurate localization, clearer boundary delineation, and improved robustness.

### 3.2 Decoder block

**FIGURE 2:**
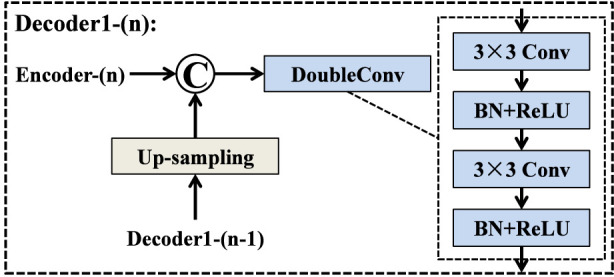
Structure of Decoder1 block.

The right-side decoder, referred to as Decoder1, follows the classical decoding strategy adopted in the original U-Net architecture. As illustrated in Figure 2, each decoding stage consists of two main operations: an up-sampling step and a feature refinement step. Specifically, the feature map from the previous decoding layer is first up-sampled to match the spatial resolution of the corresponding encoder feature. This restored feature map is then concatenated with the feature map extracted from the encoder at the same hierarchical level, allowing the decoder to integrate both high-level semantic information and fine-grained spatial details. After concatenation, the fused feature map is passed through a DoubleConv block for comprehensive feature refinement. The DoubleConv block contains two consecutive 3 × 3 convolutional layers, each followed by batch normalization and a ReLU activation function. This structure effectively enhances spatial representation, suppresses noise, and improves the decoder’s ability to reconstruct accurate boundary and region information. By progressively applying this process across all decoding stages, Decoder1 generates a detailed reconstruction of the segmentation mask, which serves as both an initial prediction and a guidance signal for Decoder2.

**FIGURE 3:**
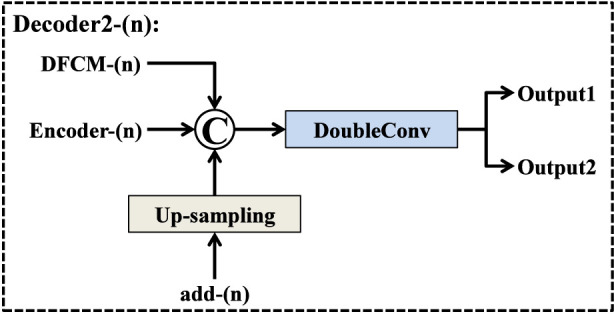
Structure of Decoder2 block.

The Decoder2 located on the left side of SECT-Net is designed as a mask-based refinement branch, aiming to further enhance the distinction between the target area and the background area. As illustrated in Figure 3, each Decoder2 block receives three types of inputs: the feature map from the previous Decoder2 level that has interacted with the mask from Decoder1, the output of the deep feature capture module at the current scale, and the corresponding encoder feature at the same resolution. Firstly, the feature map propagated from the previous Decoder2 layer is up-sampled to match the spatial size of the current decoding stage. This up-sampled feature is duplicated into two identical copies. One copy is multiplied element-wise with the resized mask generated by Decoder1 at the same scale, which enables the network to emphasize tumor related regions and suppress background responses. The other copy is then added element-wise to the mask weighted feature map, forming a residual mask interaction term denoted as add-(n). In this way, the network preserves useful contextual information while explicitly reinforcing regions that are consistent with the coarse prediction of Decoder1. The resulting add-(n) feature is then concatenated with two additional inputs: the DFCM output at level n, which provides enriched high level semantic information, and the encoder feature at the corresponding scale, which supplies fine structural details. The concatenated feature tensor is passed through a DoubleConv block that consists of two successive 3 × 3 convolutions. This operation refines the fused representations and produces the output feature maps of Decoder2 at stage n. One part of this output is forwarded to the next Decoder2 block as input for the subsequent up-sampling step, while the final Decoder2 output is used to generate the refined segmentation result. Through this multi-source fusion and mask guided refinement process, Decoder2 effectively suppresses irrelevant regions and enhances the response of true lesion areas, leading to more accurate and robust liver tumor segmentation.

### 3.3 SE-convolution transformer module

**FIGURE 4:**
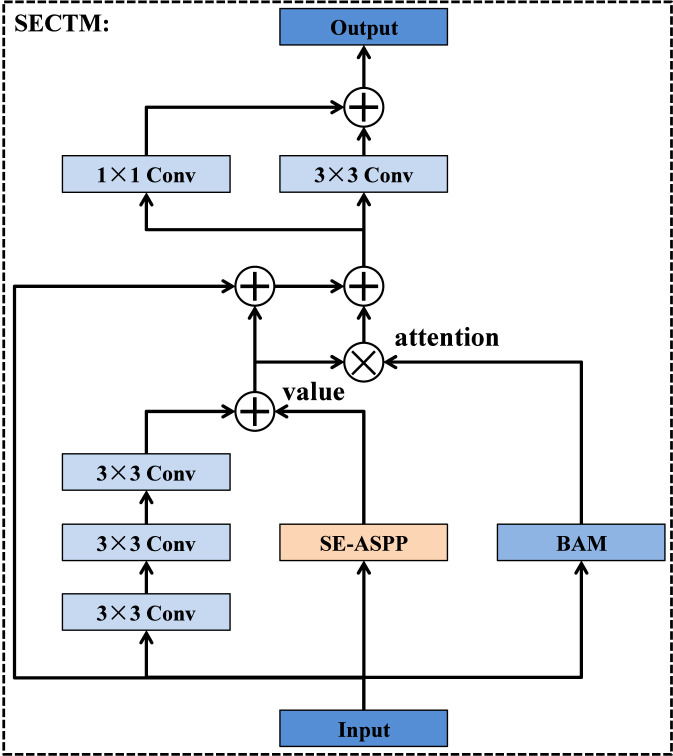
Structure of SE-convolution Transformer module.

Although the encoder in U-Net is effective in extracting local spatial patterns, its ability to model long-range dependencies is limited, especially when processing low-resolution features in deeper layers. To address this limitation, we design the SE-convolution Transformer module, as illustrated in Figure 4. Specifically, the module begins by feeding the input feature map into three parallel branches. The first branch consists of stacked 3 × 3 convolutional layers that extract rich local details and short-range spatial cues. The second branch processes the same input through the SE-ASPP block, which expands the receptive field using multiple atrous convolution rates to generate multi-scale contextual information. The outputs of these two branches are then added element-wise to form the value tensor, which integrates both detailed and multi-scale structural cues. In addition to forming the value tensor, a residual connection directly adds the original input feature map to this value representation, further enhancing feature consistency and stabilizing gradient flow. Meanwhile, the input feature maps are also forwarded into a third branch designed to compute attention weights. This branch employs a bilinear attention module (BAM), which consists of point convolution layers and fully connected layers. It generates an attention map that highlights informative regions while suppressing irrelevant or noisy responses. The resulting attention map is multiplied with the value tensor, enabling the module to selectively enhance meaningful structures based on global contextual relationships. The attended tensor is then added once more to the value tensor, producing a balanced representation that incorporates local detail, multi-scale context, and global attention cues. This fused representation is subsequently refined through two parallel stages of 3 × 3 convolutions, each further enhancing spatial consistency and reducing noise. Finally, the outputs of these refinement convolutions are added together to generate the final output of the SE-convolution Transformer module. By combining convolutional processing, multi-scale receptive field expansion, and a linear attention mechanism based on pointwise convolution and fully connected layers, the SE-convolution Transformer module significantly enhances the encoder’s ability to capture long-range dependencies while preserving fine structural information. Finally, the outputs of these two refinement branches are subsequently fused through element-wise addition to produce the final output of the SE-convolution Transformer module. By combining convolutional processing, multi-scale receptive field expansion, and a linear attention mechanism based on pointwise convolution and fully connected layers, the SE-convolution Transformer module significantly enhances the encoder’s ability to capture long-range dependencies while preserving fine structural information.

**FIGURE 5:**
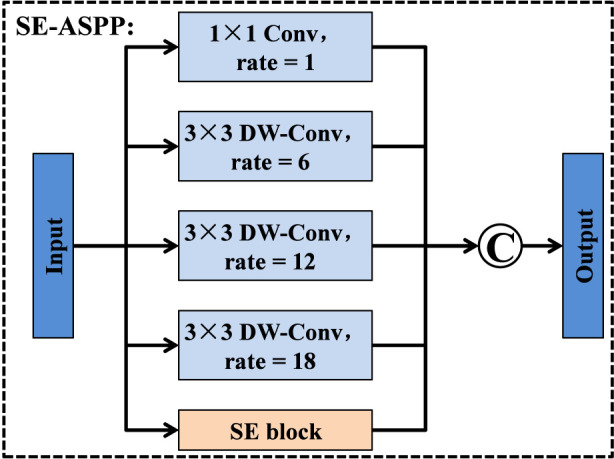
Structure of SE-ASPP module.

**FIGURE 6:**
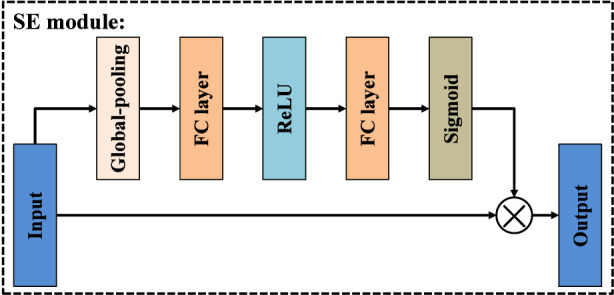
Structure of SE module.

Although standard convolutional layers can capture local spatial structures, they are often insufficient for modeling lesions that exhibit large scale variation and complex contextual relationships. To address this limitation while maintaining a manageable computational cost, we design the SE-ASPP module, which explicitly integrates the core ideas of squeeze-and-excitation (Kunjumon et al., 2025; Wang et al., 2025c) and atrous spatial pyramid pooling (ASPP) (Chen et al., 2017), as illustrated in Figure 5. This module adopts a multi-branch architecture that aggregates features at different dilation scales and incorporates channel-wise recalibration. Specifically, given an input feature map, SE-ASPP first splits it into five parallel convolutional branches. The first branch applies a 1 × 1 convolution with dilation rate 1, which preserves the original spatial resolution and focuses on refining local semantic details. The remaining three branches employ 3 × 3 depthwise convolutions (DW-Conv) with dilation rates of 6, 12, and 18, respectively. Inspired by ASPP, these dilated depthwise convolutions effectively enlarge the receptive field and capture multi-scale contextual cues without introducing a significant parameter overhead. Consequently, the four convolutional branches jointly encode both fine-grained local patterns and long-range structural information. In parallel, the input feature map is also forwarded to a SE block. As illustrated in Figure 6, the SE block first performs global spatial pooling to squeeze each channel into a single descriptor, then passes these descriptors through a small fully connected (FC) bottleneck to learn channel-wise importance weights. These weights are used to excite informative channels and suppress less relevant ones, enabling the module to adaptively emphasize discriminative feature responses. Finally, the outputs from the four convolutional branches and the SE block are concatenated along the channel dimension to form the output of the SE-ASPP module. By combining the multi-scale context modeling capability of ASPP with the adaptive channel recalibration of SE, the SE-ASPP module provides rich and discriminative contextual representations for subsequent processing in the SE-convolution Transformer module, and effectively improves the network’s ability to represent lesions with diverse sizes and irregular shapes.

**FIGURE 7:**
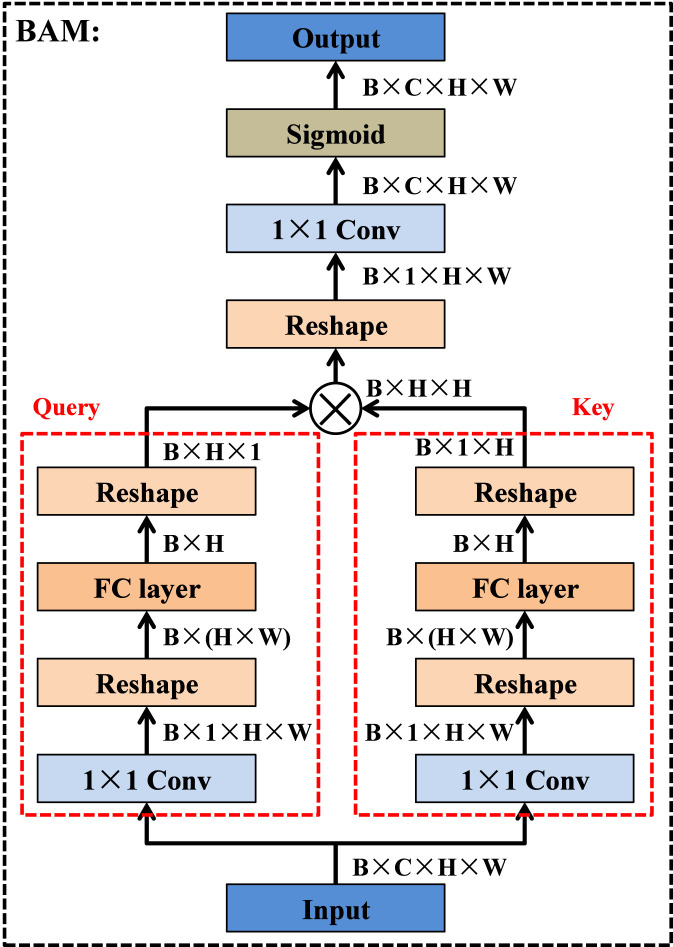
Structure of bilinear attention module.

The bilinear attention module is designed to generate an expressive attention map by modeling second-order interactions between feature representations. As illustrated in Figure 7, given an input feature map $X\in R^{B\times C\times H\times W}$ , where B denotes the batch size, C represents the number of channels, and H and W correspond to the spatial height and width, the module first transforms X into two parallel feature embeddings, namely, the query branch and the key branch. Each branch begins with a 1 × 1 convolution that projects the input into a compact feature space of size B × 1×H × W, preserving spatial resolution while performing linear channel compression. The outputs of the two 1 × 1 convolutions are then reshaped from B × 1×H × W into a vectorized representation of size B × (H × W). This flattened descriptor is fed into a fully connected layer, which reduces the feature dimension to B × H, enabling the module to learn global structural relationships across spatial positions. The resulting query and key representations, both with shape B × H, are subsequently reshaped into B × H×1 and B × 1×H, respectively. To capture second-order dependencies, these two matrices are multiplied through a bilinear interaction operation, producing a bilinear correlation matrix of size B × H × H. This operation effectively encodes pairwise spatial interactions and strengthens the module’s ability to model long-range dependencies. The bilinear matrix is then reshaped back to a spatial tensor with size B × 1×H × H, followed by another 1 × 1 convolution that adaptively integrates the learned pairwise correlations. Finally, a sigmoid activation normalizes the responses to the range [0-1], yielding the final attention map of size B × C × H × W. This attention map is subsequently applied to the value tensor in the SE-convolution Transformer module to selectively emphasize informative regions while effectively suppressing irrelevant or noisy activations.

### 3.4 Deep feature capture module

**FIGURE 8:**
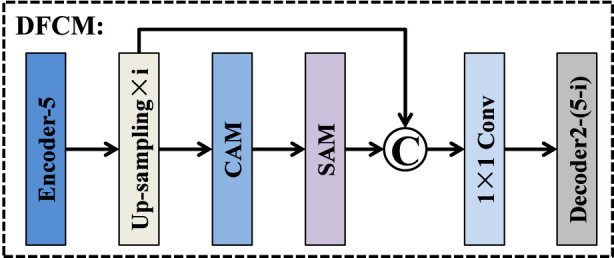
Structure of deep feature capture module.

Although the bottleneck layer of an encoder-decoder architecture contains the richest high-level semantics, these deep features are typically injected only into the final decoding stage, leading to insufficient semantic guidance for earlier decoder layers. This limitation becomes more pronounced in medical image segmentation, where accurate delineation of small, low-contrast, or ambiguous structures relies heavily on strong semantic cues across multiple spatial scales. To address this issue, we introduce a deep feature capture module that redistributes the semantic information of the bottleneck layer to all decoder stages in a scale-aware manner. As illustrated in Figure 8, the output feature map of Encoder-5 is routed into four parallel branches, each corresponding to a specific decoding layer. In the *i*th branch (i = 1, 2, 3, 4), the deep feature map is progressively up-sampled i times to match the spatial resolution of its target decoder layer Decoder2-(5-i). After spatial alignment, each branch sequentially employs a channel attention module (CAM) (Cai et al., 2025; He et al., 2024) and a spatial attention module (SAM) (Fu et al., 2023; Zhang et al., 2023) to enhance discriminative channel responses and emphasize salient spatial regions. A residual connection from Up-sampling × i is then fused with the attention-enhanced features to maintain semantic consistency. The fused representation is subsequently compressed and refined via a 1 × 1 convolution, resulting in a compact yet informative guidance feature. Finally, the outputs of the four branches are fed into their corresponding decoder layers, enabling multi-scale semantic reinforcement throughout the decoding process. Unlike existing methods, by integrating semantic redistribution, global guidance, and attention refinement into a single pre-decoding module, the DFCM significantly improves feature reconstruction and enhances the delineation of complex tumor boundaries.

**FIGURE 9:**
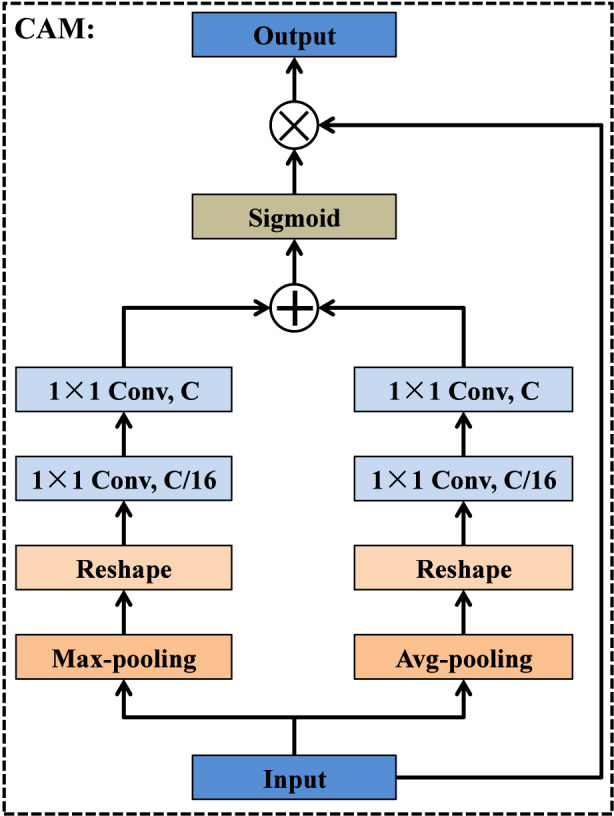
Structure of channel attention module.

As illustrated in Figure 9, the channel attention module aims to adaptively reweight channel-wise feature responses by exploiting both max-pooling and average-pooling priors. Given an input feature map, CAM first generates two global descriptors by applying max-pooling and average-pooling operations across the spatial dimensions. Each descriptor is then reshaped and passed through a shared two-layer transformation consisting of a 1 × 1 convolution that reduces the channel dimension to C/16, followed by another 1 × 1 convolution that restores it back to C. The two transformed descriptors are subsequently aggregated through element-wise addition and activated by a sigmoid function to produce a channel attention map. Finally, this attention map is multiplied with the original input feature map, enabling the network to emphasize informative channels while suppressing irrelevant or noisy ones.

**FIGURE 10:**
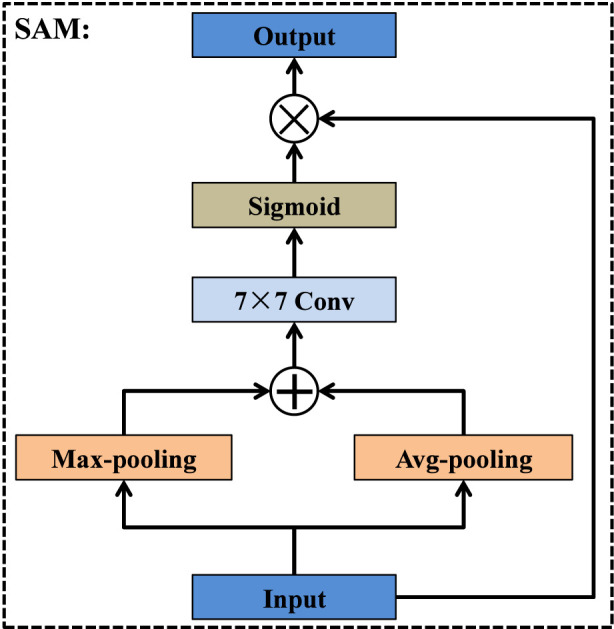
Structure of spatial attention module.

As shown in Figure 10, the spatial attention module focuses on enhancing salient spatial regions by aggregating complementary spatial cues from the input feature map. Given the input, SAM first applies max-pooling and average-pooling operations along the channel dimension to generate two spatial descriptors, each of size H × W. These descriptors capture distinct spatial statistics and are subsequently fused through element-wise addition. The combined representation is then processed by a 7 × 7 convolution layer, which enlarges the receptive field and enables the module to integrate broader contextual information. A sigmoid activation is applied to the convolution output to produce the spatial attention map, which highlights informative spatial locations. Finally, the attention map is multiplied with the original input feature map to adaptively emphasize relevant regions while suppressing background noise.

### 3.5 Loss function

In liver tumor segmentation, a major difficulty lies in the extremely uneven distribution of foreground and background pixels. Additionally, liver tumors frequently present vague boundaries, heterogeneous internal intensities, and significant variability in shape, all of which further reduce the effectiveness of losses that rely solely on pixel-level accuracy. To address these challenges, the Dice loss provides a more suitable optimization objective because it evaluates the similarity between prediction and ground truth on a regional basis rather than counting individual pixels. For these reasons, the Dice loss is selected to guide network training, as defined in Equation 1:

$$L_{Dice}=1-\frac{2\sum_{i=1}^{N}y_{i}{\hat{y}}_{i}}{\sum_{i=1}^{N}y_{i}^{2}+\sum_{i=1}^{N}{\hat{y}}_{i}^{2}}$$ (1)

where N is the number of pixels, ${\hat{y}}_{i}$ and $y_{i}$ denote the labeled value and predicted value.

## 4 Experiments

### 4.1 Dataset

**FIGURE 11:**
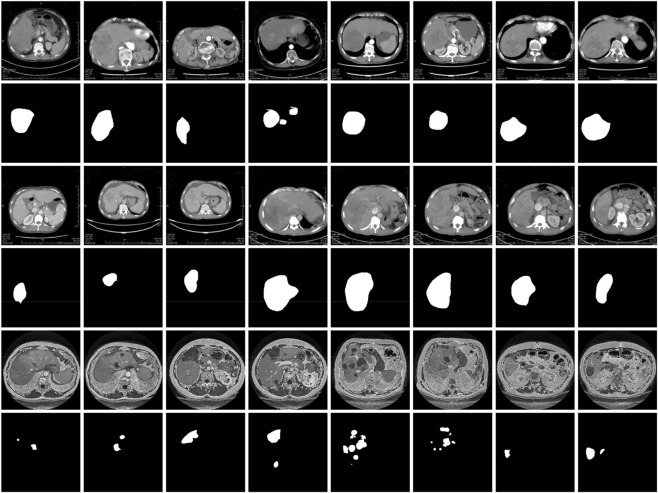
Illustrative examples from the arterial phase, portal venous phase, and 3DIRCADb datasets. The first two rows display representative arterial phase images alongside their corresponding ground-truth annotations, the third and fourth rows provide comparable examples from the portal venous phase dataset, while the last two rows are examples from the 3DIRCADb dataset.

To rigorously evaluate the performance of SECT-Net, we employed two contrast-enhanced CT datasets collected from the Infectious Diseases Department of Quzhou People’s Hospital, corresponding to the arterial phase and the portal venous phase, and further assessed its generalizability on the publicly available 3DIRCADb dataset. As illustrated in Figure 11, representative samples from the arterial phase dataset, the portal venous-phase dataset, and the 3DIRCADb dataset are provided for visual comparison. Considering the computational complexity of the SECT-Net framework and the memory limitations of the available hardware, all CT slices were resampled to a unified spatial resolution of 256 × 256 pixels to ensure stable training and consistent input representation. All datasets were strictly partitioned at the patient level, ensuring that images from the same patient were never shared across the training, validation, and testing sets. Specifically, no images or slices from a single patient were partially used for training while the remaining data were assigned to validation or testing, thereby preventing information leakage and enabling a realistic evaluation of model performance on previously unseen patients. For the arterial phase dataset, a total of 2,112 images were included, comprising 1,268 images for training, 422 for validation, and 422 for testing. Similarly, the portal venous phase dataset contained 2,103 images, which were divided into 1,263 training samples, 420 validation samples, and 420 testing samples. For the 3DIRCADb dataset (Wang Z. et al., 2025), we utilized a total of 2,823 images, which were split into 1,695 for training, 564 for validation, and 564 for testing. A consolidated summary of these dataset configurations and their respective partitions is presented in Table 1.

**TABLE 1:** Detailed overview of the arterial phase, portal venous phase, and 3DIRCADb datasets.

Dataset	Number	Training	Validation	Testing
Arterial phase	2112	1268	422	422
Portal venous phase	2103	1263	420	420
3DIRCADb	2,823	1,695	564	564

### 4.2 Evaluation metrics

To thoroughly assess the segmentation capability of SECT-Net and to enable a fair comparison with established baseline methods, three commonly adopted quantitative metrics were utilized: Dice (Wan et al., 2025a; Li et al., 2022), Mcc (Wan et al., 2025b; Shang et al., 2024), and Jaccard (Yang et al., 2024; Yuan et al., 2024). The Dice, Mcc, and Jaccard metrics are defined in Equations 2–4:

$$Dice=\frac{2TP}{2TP+FN+FP}$$ (2)

$$Mcc=\frac{TP\times TN-FP\times FN}{\sqrt{\left(TP+FN\right)\left(TP+FP\right)\left(TN+FN\right)\left(TN+FP\right)}}$$ (3)

$$Jaccard=\frac{TP}{TP+FN+FP}$$ (4)

### 4.3 Parameter setting

The experimental implementation of SECT-Net was carried out on a 64-bit windows workstation, with the entire framework developed using the PyTorch 2.0.0 deep learning platform. All training and inference procedures were executed on an NVIDIA GeForce RTX 4090 GPU, which provides 24 GB of dedicated memory. To ensure stable optimization and reliable convergence, several training hyperparameters were carefully selected. The mini-batch size was configured as 16 to balance memory usage and gradient stability, while the initial learning rate was set to 0.001. The training process was executed for 200 iterations, allowing the model to sufficiently learn complex tumor representations. Moreover, the Adam optimizer was employed owing to its adaptive moment estimation and proven effectiveness in accelerating convergence.

**FIGURE 12:**
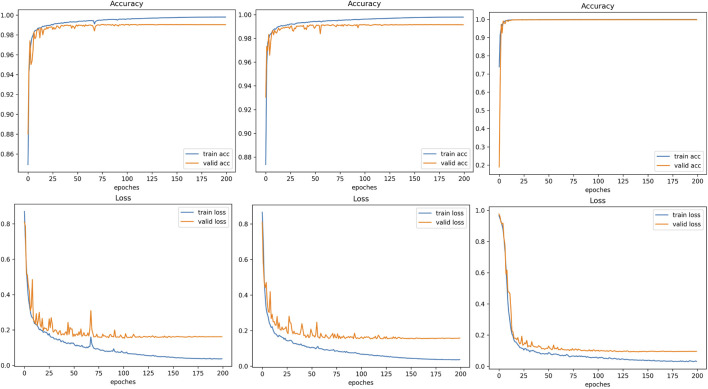
The training dynamics of SECT-Net on the arterial phase, portal venous phase, and 3DIRCADb datasets. The plots in the first column depict the evolution of accuracy and loss throughout the training process for the arterial phase images, the second column presents the corresponding curves generated from the portal venous phase dataset, while the third column are the corresponding curves generated from the 3DIRCADb dataset.

Figure 12 illustrates the learning dynamics of SECT-Net on the arterial-phase, portal venous-phase, and 3DIRCADb datasets. From left to right, the three columns correspond to the arterial-phase dataset, the portal venous-phase dataset, and the 3DIRCADb dataset, respectively. For all three datasets, the accuracy curves exhibit a rapid increase during the early training epochs and gradually stabilize as the model converges. The training accuracy approaches near-saturation, while the validation accuracy remains consistently high after a short warm-up period, indicating strong generalization capability across different data distributions. Meanwhile, the loss curves show a steady downward trend. Although minor fluctuations are observed in the validation loss during the initial training stage, they gradually diminish and converge to relatively low values. Overall, these curves demonstrate that SECT-Net can efficiently learn discriminative tumor representations and achieve stable convergence without noticeable overfitting on any of the three datasets.

### 4.4 Ablation experiments

Table 2 presents the ablation experiments conducted on the arterial phase dataset to examine how each component contributes to the final performance of SECT-Net. The study begins with a standard U-Net configuration, which serves as the foundational model for comparison. When the dual-decoder structure is incorporated into this baseline, a slight improvement is observed in all evaluation metrics, reflecting the benefit of employing two decoding pathways to enrich feature restoration and better recover tumor regions. On top of the dual-decoder configuration, integrating the SE-convolution Transformer module produces a notable performance boost. This outcome suggests that the joint use of convolutional operations and Transformer-style global modeling helps capture complementary contextual cues that are essential for precise segmentation. Introducing the deep feature capture module into the dual-decoder network also enhances segmentation accuracy, although to a lesser extent compared with SECTM. This enhancement indicates that propagating multi-scale semantic information from the bottleneck layer to different decoding stages supports more effective feature refinement. The full SECT-Net, which combines the dual-decoder structure with both SECTM and DFCM, achieves the highest scores across all metrics. This result confirms that the two modules contribute distinct advantages and that their interaction further strengthens the network’s ability to model complex liver tumor patterns. Overall, the ablation findings demonstrate that each component plays an important role, and the complete SECT-Net configuration provides the most comprehensive improvements in segmentation quality.

**TABLE 2:** Ablation experiments of SECT-Net on the arterial phase dataset.

Method	Dice	Mcc	Jaccard
Baseline (U-Net)	0.8236 ± 0.0441	0.8203 ± 0.0424	0.7025 ± 0.0642
Baseline + Dual-decoder	0.8274 ± 0.0464	0.8242 ± 0.0441	0.7083 ± 0.0668
Baseline + Dual-decoder + SECTM	0.8446 ± 0.0382	0.8408 ± 0.0367	0.7329 ± 0.0564
Baseline + Dual-decoder + DFCM	0.8325 ± 0.0408	0.8288 ± 0.0388	0.7151 ± 0.0592
SECT-Net	0.8452 ± 0.0394	0.8411 ± 0.0384	0.7339 ± 0.0575

To further investigate the effectiveness of the proposed mask-guided mechanism within the dual-decoder framework of SECT-Net, we conducted an ablation experiment on the arterial-phase dataset, as reported in Table 3. Specifically, we constructed a comparative variant, denoted as SECT-Net with Decoder1-guided, in which the mask-guided interaction was replaced by direct feature transfer. That is, the intermediate features from Decoder1-2, Decoder1-3, and Decoder1-4 were directly fused into the corresponding layers of Decoder2 without using the coarse segmentation mask produced by Decoder1 as an explicit guidance signal. The Decoder1-guided variant achieves Dice, Mcc, and Jaccard scores of 0.8309, 0.8271, and 0.7128, whereas incorporating the proposed mask-guided strategy consistently improves the performance to 0.8452, 0.8411, and 0.7339. These results indicate that simply transferring decoder features yields limited gains, while leveraging a coarse segmentation mask as a structural prior provides more effective guidance for feature refinement, enabling the second decoder to suppress background interference and progressively enhance boundary delineation. Overall, this ablation study demonstrates that the mask-guided interaction is a key factor in fully exploiting the advantages of the dual-decoder architecture in SECT-Net, leading to more discriminative representations and improved segmentation accuracy.

**TABLE 3:** Ablation experiments of mask-guided in SECT-Net on the arterial phase dataset.

Method	Dice	Mcc	Jaccard
SECT-Net with Decoder1-guided	0.8309 ± 0.0411	0.8271 ± 0.0387	0.7128 ± 0.0591
SECT-Net with mask-guided	0.8452 ± 0.0394	0.8411 ± 0.0384	0.7339 ± 0.0575

To further investigate the contributions of the proposed SE-ASPP module and BAM within the SECTM framework, we conducted ablation experiments on the arterial-phase dataset, as reported in Table 4. Specifically, we constructed several comparative variants by replacing SE-ASPP with its individual components or removing key modules to evaluate their respective effects. Quantitative results show that SECTM with SE achieves Dice, Mcc, and Jaccard scores of 0.8194, 0.8162, and 0.6970, while SECTM with ASPP improves the performance to 0.8393, 0.8354, and 0.7250. However, both remain inferior to the complete SECTM, which achieves the best performance with Dice, Mcc, and Jaccard scores of 0.8452, 0.8411, and 0.7339. Although ASPP alone is more effective than SE alone due to its ability to capture multi-scale contextual information, neither mechanism is sufficient by itself. Notably, removing the SE-ASPP module entirely leads to the most pronounced performance degradation, with the Dice score dropping to 0.8134 and consistent declines in Mcc and Jaccard, indicating that the structured integration of channel-wise recalibration and multi-scale context aggregation plays a central role in enhancing feature representations. In contrast, removing BAM causes only a slight performance decline, suggesting that BAM provides complementary refinement rather than serving as the primary performance driver. Overall, this ablation study demonstrates that the performance gains of SECTM mainly stem from the synergistic integration embodied in the SE-ASPP module, while BAM further enhances segmentation performance in a complementary manner.

**TABLE 4:** Ablation experiments of SE-ASPP and BAM in SECTM on the arterial phase dataset.

Method	Dice	Mcc	Jaccard
SECTM with SE	0.8194 ± 0.0497	0.8162 ± 0.0474	0.6970 ± 0.0707
SECTM with ASPP	0.8393 ± 0.0388	0.8354 ± 0.0377	0.7250 ± 0.0574
SECTM without SE-ASPP	0.8134 ± 0.0460	0.8095 ± 0.0443	0.6880 ± 0.0646
SECTM without BAM	0.8395 ± 0.0403	0.8360 ± 0.0389	0.7255 ± 0.0590
SECTM	0.8452 ± 0.0394	0.8411 ± 0.0384	0.7339 ± 0.0575

### 4.5 Comparison experiments

Table 5 provides a detailed quantitative comparison of several representative liver tumor segmentation networks on the arterial phase dataset. The results reveal that SECT-Net delivers the highest performance across all three evaluation metrics, achieving Dice, Mcc, and Jaccard scores of 0.8452, 0.8411, and 0.7339, respectively. These values outperform all other competing models, underscoring the effectiveness of SECT-Net in capturing multi-scale contextual information and delineating lesion boundaries in arterial phase CT images. Among them, NLIE-UNet attains the best results (Dice = 0.8234, Mcc = 0.8197, Jaccard = 0.7021), benefiting from non-local interactions that enhance contextual aggregation. LMFR-Net (Dice = 0.8006, Mcc = 0.7966, Jaccard = 0.6706) and MAUNet (Dice = 0.8150, Mcc = 0.8107, Jaccard = 0.6897) follow, both relying on enhanced encoder-decoder pathways but offering limited long-range modeling capability compared to SECT-Net. MBSNet achieves the strongest results within this group (Dice = 0.8250, Mcc = 0.8215, Jaccard = 0.7040), demonstrating the advantage of multi-branch feature selection. DDANet (Dice = 0.8104, Mcc = 0.8071, Jaccard = 0.6840) and TRFENet (Dice = 0.8049, Mcc = 0.8007, Jaccard = 0.6765) also provide reasonable segmentation accuracy, but their dual-path feature fusion strategies do not fully capture the complex appearance variations of arterial phase tumors. Transformer-based models, including PVTFormer, SwinT, and TransUNet, show comparatively lower performance. SwinT leads this category with a Dice of 0.8058, attributable to its hierarchical self-attention mechanism. PVTFormer (Dice = 0.7922, Mcc = 0.7874, Jaccard = 0.6585) shows slightly lower accuracy, likely due to insufficient fine-grained feature preservation in the shallow layers. In particular, TransUNet achieves Dice, Mcc, and Jaccard scores of 0.7542, 0.7494, and 0.6093, indicating that directly integrating transformer mechanism into U-Net architectures may be insufficient for preserving fine-grained spatial details in arterial phase liver tumor segmentation. Overall, SECT-Net consistently outperforms all eight competing methods. Its improvements over the strongest baseline (MBSNet) reach 2.02% in Dice, 1.96% in Mcc, and 2.99% in Jaccard, demonstrating superior segmentation accuracy and more stable convergence behavior on the arterial phase liver tumor dataset.

**TABLE 5:** Comparison experiment on the arterial phase dataset.

Method	Dice	Mcc	Jaccard
LMFR-Net (Zhang et al., 2025)	0.8006 ± 0.0516	0.7966 ± 0.0500	0.6706 ± 0.0714
NLIE-UNet (Wan et al., 2025c)	0.8234 ± 0.0444	0.8197 ± 0.0418	0.7021 ± 0.0633
MAUNet (Li et al., 2025)	0.8150 ± 0.0401	0.8107 ± 0.0395	0.6897 ± 0.0581
TRFENet (Gong et al., 2023)	0.8049 ± 0.0512	0.8007 ± 0.0489	0.6765 ± 0.0699
MBSNet (Jin et al., 2023)	0.8250 ± 0.0393	0.8215 ± 0.0373	0.7040 ± 0.0567
DDANet (Tomar et al., 2021)	0.8104 ± 0.0482	0.8071 ± 0.0452	0.6840 ± 0.0682
PVTFormer (Jha et al., 2024)	0.7922 ± 0.0488	0.7874 ± 0.0487	0.6585 ± 0.0667
SwinT (Xu et al., 2024)	0.8058 ± 0.0482	0.8013 ± 0.0471	0.6775 ± 0.0681
TransUNet (Chen et al., 2021)	0.7542 ± 0.0616	0.7494 ± 0.0609	0.6093 ± 0.0790
SECT-Net	0.8452 ± 0.0394	0.8411 ± 0.0384	0.7339 ± 0.0575

**FIGURE 13:**
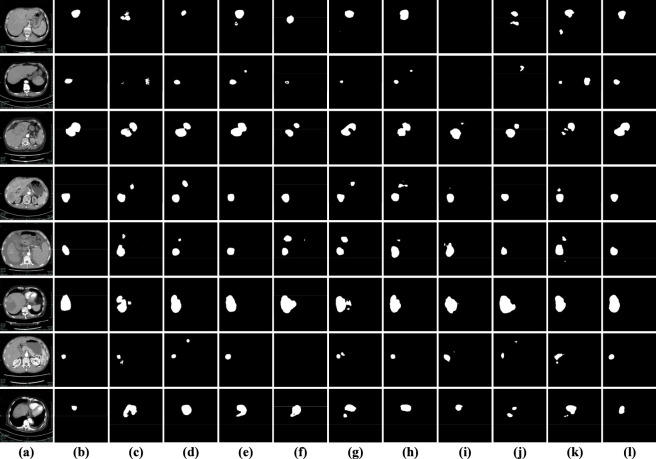
Visual results of all models on the arterial phase dataset. **(a)** original images; **(b)** labeled images; **(c)** LMFR-Net; **(d)** NLIE-UNet; **(e)** MAUNet; **(f)** TRFENet; **(g)** MBSNet; **(h)** DDANet; **(i)** PVTFormer; **(j)** SwinT; **(k)** TransUNet; **(l)** SECT-Net.

Figure 13 illustrates the visual results of all competing models on the arterial phase liver tumor dataset. The first column displays the original CT images, followed by the corresponding ground truth masks, while the remaining columns present the predictions generated by LMFR-Net, NLIE-UNet, MAUNet, TRFENet, MBSNet, DDANet, PVTformer, SwinT, TransUNet, and the proposed SECT-Net. Visually, most of the methods are able to detect the main tumor regions. However, noticeable differences emerge in terms of boundary integrity, lesion completeness, and robustness to intensity variations. SECT-Net produces the most precise and stable segmentation, yielding smooth and continuous boundaries and successfully capturing both large tumors and small peripheral lesions. Transformer-based models such as PVTFormer and SwinT demonstrate strong global perception ability and are effective in capturing the overall tumor structure, but they occasionally exhibit over-segmentation or boundary leakage in low-contrast or heterogeneous enhancement regions. TransUNet, which combines a transformer encoder with a U-Net-style decoder, shows improved global contextual modeling compared with pure CNN-based methods. However, it still struggles to preserve fine-grained spatial details, often resulting in blurred boundaries and partial omission of small or irregular lesions in arterial phase images. Methods including MBSNet, TRFENet, and MAUNet achieve moderate visual performance, accurately outlining the primary tumor mass but showing reduced sensitivity to subtle lesion regions and complex boundary variations. In contrast, LMFR-Net, NLIE-UNet, and DDANet exhibit weaker visual consistency, frequently producing fragmented predictions, under-segmentation, or blurred contours, particularly in slices with pronounced intensity heterogeneity. These qualitative observations are well aligned with the quantitative results reported in Table 5, demonstrating that SECT-Net achieves superior feature representation and boundary-aware segmentation performance for arterial phase liver tumor images.

**FIGURE 14:**
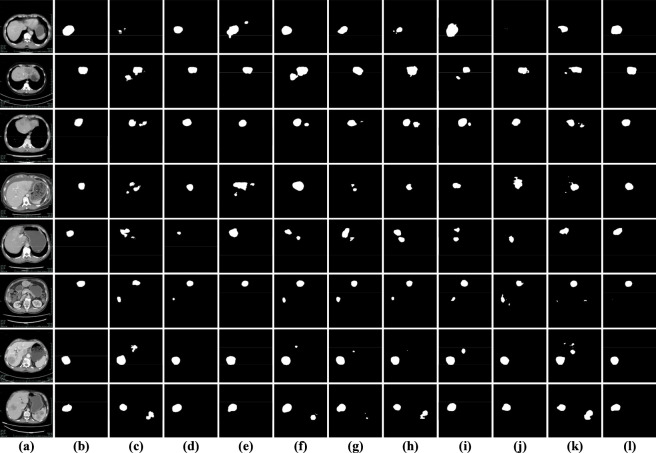
Visual results of all models on the portal venous phase dataset. **(a)** original images; **(b)** labeled images; **(c)** LMFR-Net; **(d)** NLIE-UNet; **(e)** MAUNet; **(f)** TRFENet; **(g)** MBSNet; **(h)** DDANet; **(i)** PVTFormer; **(j)** SwinT; **(k)** TransUNet; **(l)** SECT-Net.

As reported in Table 6, SECT-Net achieves the best overall performance on the portal venous phase dataset, reaching Dice = 0.8425, Mcc = 0.8396, and Jaccard = 0.7339, outperforming all competing models. Compared with the second-best MBSNet, SECT-Net delivers additional gains of 0.30%, 0.31%, and 0.47% on Dice, Mcc, and Jaccard, respectively. Although the margin appears moderate, this improvement reflects SECT-Net’s enhanced ability to model multi-scale contextual cues and maintain accurate boundary localization. When compared with transformer-based models, including PVTFormer, SwinT, and TransUNet, SECT-Net demonstrates clear advantages. Specifically, relative to TransUNet, SECT-Net improves Dice, Mcc, and Jaccard by 4.98%, 4.90%, and 6.74%, respectively. While TransUNet benefits from transformer-driven global context modeling, its heavy reliance on patch-wise self-attention often leads to insufficient preservation of fine-grained spatial details, resulting in blurred boundaries and incomplete segmentation of small or irregular tumors in the portal venous phase. Similarly, PVTFormer and SwinT show strong global perception capabilities but suffer from fragmented predictions or missed lesion regions under low-contrast conditions. Among CNN-based methods, MBSNet ranks second and produces relatively compact segmentation masks. However, slight boundary discontinuities and local inaccuracies are still observed. NLIE-UNet, MAUNet, and TRFENet deliver moderate performance, accurately identifying major tumor regions but frequently under-segmenting small nodules or introducing isolated noisy regions. LMFR-Net and DDANet exhibit comparatively weaker robustness, particularly in cases with heterogeneous enhancement patterns, where lesion details are either oversmoothed or partially suppressed. The qualitative visual comparisons in Figure 14 further support these quantitative findings. SECT-Net consistently generates clean, complete, and well-shaped tumor masks with smooth and continuous boundaries that closely align with the ground truth annotations. Both compact and irregular lesions are accurately captured, with minimal false positives or leakage into surrounding tissues. Overall, the combined quantitative and qualitative results demonstrate that SECT-Net delivers the most reliable and precise segmentation performance on the portal venous phase dataset, highlighting its superior multi-scale feature modeling capability and enhanced boundary awareness across diverse tumor appearances.

**TABLE 6:** Comparison experiment on the portal venous phase dataset.

Method	Dice	Mcc	Jaccard
LMFR-Net (Zhang et al., 2025)	0.8114 ± 0.0823	0.8080 ± 0.0807	0.6898 ± 0.1030
NLIE-UNet (Wan et al., 2025b)	0.8353 ± 0.0703	0.8323 ± 0.0688	0.7228 ± 0.0929
MAUNet (Li et al., 2025)	0.8269 ± 0.0737	0.8250 ± 0.0712	0.7110 ± 0.0983
TRFENet (Gong et al., 2023)	0.8222 ± 0.0756	0.8195 ± 0.0750	0.7044 ± 0.0997
MBSNet (Jin et al., 2023)	0.8395 ± 0.0704	0.8365 ± 0.0687	0.7292 ± 0.0959
DDANet (Tomar et al., 2021)	0.8116 ± 0.0902	0.8078 ± 0.0898	0.6914 ± 0.1124
PVTFormer (Jha et al., 2024)	0.7944 ± 0.0887	0.7914 ± 0.0872	0.6672 ± 0.1138
SwinT (Xu et al., 2024)	0.7850 ± 0.0852	0.7809 ± 0.0842	0.6535 ± 0.1049
TransUNet (Chen et al., 2021)	0.7927 ± 0.1030	0.7906 ± 0.0968	0.6665 ± 0.1168
SECT-Net	0.8425 ± 0.0732	0.8396 ± 0.0721	0.7339 ± 0.0977

**FIGURE 15:**
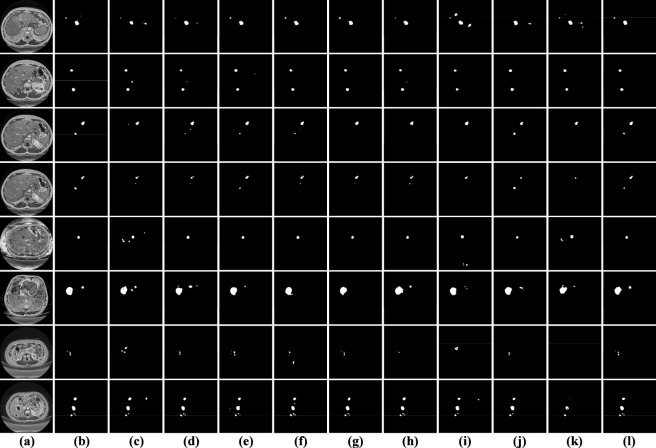
Visual results of all models on the 3DIRCADb dataset. **(a)** original images; **(b)** labeled images; **(c)** LMFR-Net; **(d)** NLIE-UNet; **(e)** MAUNet; **(f)** TRFENet; **(g)** MBSNet; **(h)** DDANet; **(i)** PVTFormer; **(j)** SwinT; **(k)** TransUNet; **(l)** SECT-Net.

As summarized in Table 7, SECT-Net delivers the highest accuracy on the 3DIRCADb dataset, achieving Dice = 0.8845, Mcc = 0.8855, and Jaccard = 0.7969. In comparison with the second-ranked MBSNet, SECT-Net shows incremental gains of 0.29%, 0.36%, and 0.60% on the three metrics, demonstrating its stronger capability in maintaining lesion integrity and refining boundary precision. Notably, when evaluated against transformer-based approaches such as PVTFormer, SwinT, and TransUNet, SECT-Net exhibits more pronounced advantages, particularly in terms of Jaccard, indicating its superior handling of small, low-contrast, and irregularly shaped tumors. The visual comparisons presented in Figure 15 further corroborate these quantitative observations. SECT-Net produces segmentation masks that are more compact, complete, and structurally consistent, effectively covering both small nodules and complex lesion shapes. Its predicted contours closely follow the ground truth, with fewer spurious responses and reduced background interference. In contrast, PVTFormer, SwinT and TransUNet frequently fail to recover subtle lesion regions or generate fragmented outputs when boundaries are ambiguous. Other CNN-based methods, including LMFR-Net, NLIE-UNet, MAUNet, and TRFENet, yield relatively reasonable predictions but tend to miss tiny lesions or introduce isolated noise. DDANet exhibits less stable behavior under challenging conditions, sometimes suppressing meaningful tumor structures. Although MBSNet achieves competitive performance and ranks second quantitatively, its results occasionally show discontinuities along lesion margins. Overall, both the numerical evaluation and visual evidence demonstrate that SECT-Net offers the most reliable and precise segmentation on the 3DIRCADb dataset, benefiting from its enhanced multi-scale representation and boundary-aware strategy.

**TABLE 7:** Comparison experiment on the 3DIRCADb dataset.

Method	Dice	Mcc	Jaccard
LMFR-Net (Zhang et al., 2025)	0.8582 ± 0.0410	0.8579 ± 0.0407	0.7538 ± 0.0624
NLIE-UNet (Wan et al., 2025a)	0.8575 ± 0.0603	0.8588 ± 0.0576	0.7553 ± 0.0902
MAUNet (Li et al., 2025)	0.8728 ± 0.0603	0.8736 ± 0.0579	0.7792 ± 0.0909
TRFENet (Gong et al., 2023)	0.8606 ± 0.0676	0.8611 ± 0.0664	0.7614 ± 0.1017
MBSNet (Jin et al., 2023)	0.8816 ± 0.0436	0.8819 ± 0.0426	0.7909 ± 0.0692
DDANet (Tomar et al., 2021)	0.8474 ± 0.0863	0.8488 ± 0.0839	0.7446 ± 0.1244
PVTFormer (Jha et al., 2024)	0.8074 ± 0.0476	0.8081 ± 0.0472	0.6796 ± 0.0671
SwinT (Xu et al., 2024)	0.8764 ± 0.0399	0.8758 ± 0.0394	0.7823 ± 0.0632
TransUNet (Chen et al., 2021)	0.7871 ± 0.0845	0.7883 ± 0.0847	0.6568 ± 0.1118
SECT-Net	0.8845 ± 0.0530	0.8855 ± 0.0502	0.7969 ± 0.0826

### 4.6 Computational efficiency

As presented in Table 8, the evaluated models exhibit substantial variation in computational complexity and inference efficiency. LMFR-Net demonstrates the highest efficiency, requiring only 3.49 GFLOPs and 0.07M parameters, and achieving an exceptional 337.78 FPS, which reflects its extremely lightweight architecture. MBSNet also maintains a low computational cost (7.60 GFLOPs, 3.81M parameters) while attaining a high inference speed of 308.38 FPS, indicating an effective balance between model compactness and operational speed. Models such as NLIE-UNet and SwinT fall into the moderate-complexity category, with approximately seven to eight GFLOPs and 3–4M parameters, corresponding to moderate inference speeds in the range of 112–114 FPS. DDANet, with 20.87 GFLOPs and 6.84M parameters, achieves 185.04 FPS, suggesting that its increased complexity provides additional representational capacity while maintaining acceptable efficiency. In contrast, MAUNet and TRFENet incur significantly higher computational burdens. MAUNet requires 48.99 GFLOPs and 17.04M parameters, while TRFENet reaches 92.39 GFLOPs and 43.24M parameters, substantially increasing memory consumption and limiting suitability for real-time or resource-constrained environments. TransUNet exhibits a markedly higher parameter count (66.82M) and computational cost (32.55 GFLOPs) compared with most CNN-based approaches, resulting in a relatively moderate inference speed of 125.02 FPS. PVTFormer, the most computationally demanding model (103.95 GFLOPs), still delivers 199.69 FPS, highlighting the parallelizability of transformer-based operations but at the cost of considerable overhead. SECT-Net exhibits a moderate-to-high computational footprint (85.16 GFLOPs, 9.72M parameters) and achieves a stable inference rate of 150.34 FPS. Although not as lightweight as LMFR-Net or MBSNet, SECT-Net provides a favorable trade-off between accuracy and efficiency.

**TABLE 8:** Computational efficiency on the arterial phase dataset.

Method	GFLOPs	Params(M)	FPS
LMFR-Net (Zhang et al., 2025)	3.49	0.07	337.78
NLIE-UNet (Wan et al., 2025c)	7.17	4.05	112.64
MAUNet (Li et al., 2025)	48.99	17.04	92.54
TRFENet (Gong et al., 2023)	92.39	43.24	205.98
MBSNet (Jin et al., 2023)	7.60	3.81	308.38
DDANet (Tomar et al., 2021)	20.87	6.84	185.04
PVTFormer (Jha et al., 2024)	103.95	5.08	199.69
SwinT (Xu et al., 2024)	7.38	2.78	113.97
TransUNet (Chen et al., 2021)	32.55	66.82	125.02
SECT-Net	85.16	9.72	150.34

### 4.7 Optimizer selection

To evaluate the influence of different optimization methods on the training stability and segmentation performance of SECT-Net, six widely used optimizers were compared on the arterial phase dataset. The results presented in Table 9 show that the choice of optimizer has a clear impact on the final accuracy. Among all tested methods, Adam provides the best overall performance, achieving Dice score of 0.8452, Mcc value of 0.8411, and Jaccard index of 0.7339. This indicates that Adam offers a favorable balance between gradient stability and convergence efficiency. NAdam shows very similar performance and ranks second, benefiting from its use of Nesterov momentum. Adamax, AdamW, and Rprop also provide competitive results, although their accuracy is slightly lower than that of Adam and NAdam. In contrast, RMSprop and SGD yield the lowest metrics, suggesting slower convergence and weaker adaptability to complex liver tumor characteristics. Overall, the comparison clearly shows that adaptive momentum-based optimizers such as Adam and NAdam are more suitable for SECT-Net. They provide higher accuracy, better gradient stability, and more reliable convergence behavior on the arterial phase dataset.

**TABLE 9:** Optimizer selection on the arterial phase dataset.

Optimizer	Dice	Mcc	Jaccard
Adamax	0.8426 ± 0.0404	0.8386 ± 0.0391	0.7300 ± 0.0589
AdamW	0.8385 ± 0.0368	0.8348 ± 0.0352	0.7236 ± 0.0542
NAdam	0.8444 ± 0.0415	0.8407 ± 0.0398	0.7329 ± 0.0602
RMSprop	0.8302 ± 0.0410	0.8262 ± 0.0392	0.7117 ± 0.0589
Rprop	0.8395 ± 0.0398	0.8358 ± 0.0382	0.7255 ± 0.0586
SGD	0.8342 ± 0.0415	0.8304 ± 0.0397	0.7178 ± 0.0604
Adam	0.8452 ± 0.0394	0.8411 ± 0.0384	0.7339 ± 0.0575

### 4.8 Selection of loss function

To analyze the influence of different loss functions on segmentation performance, we evaluated four commonly used loss functions with the quantitative results summarized in Table 10. Among them, Dice loss achieves the best overall performance, obtaining the highest Dice, Mcc, and Jaccard of 0.8452, 0.8411, and 0.7339. This advantage stems from its region-overlap–based optimization objective, which directly maximizes the agreement between predicted masks and ground-truth annotations, making it particularly suitable for liver tumor segmentation tasks characterized by severe class imbalance. BCE loss also yields competitive results, with a Dice score of 0.8378, but its pixel-wise formulation lacks explicit structural awareness, leading to slightly inferior boundary delineation. In contrast, Hausdorff loss, which focuses on minimizing boundary distance, achieves moderate performance (Dice = 0.7630), indicating that boundary constraints alone are insufficient for robust region-level segmentation. Tversky loss exhibits the lowest performance, suggesting that its asymmetric penalty design is less effective under the current dataset characteristics. Overall, these results confirm that Dice loss provides the most stable and effective optimization objective for SECT-Net, and it is therefore adopted as the default loss function in all subsequent experiments.

**TABLE 10:** Loss function on the arterial phase dataset.

Loss function	Dice	Mcc	Jaccard
Tversky	0.5195 ± 0.1062	0.5698 ± 0.0842	0.3582 ± 0.1024
Hausdorff	0.7630 ± 0.0529	0.7633 ± 0.0513	0.6198 ± 0.0690
BCE	0.8378 ± 0.0380	0.8338 ± 0.0365	0.7226 ± 0.0552
Dice	0.8452 ± 0.0394	0.8411 ± 0.0384	0.7339 ± 0.0575

## 5 Conclusion

In this study, we present SECT-Net, a segmentation framework tailored for accurate and reliable liver tumor delineation across multi-phase CT imaging. SECT-Net is constructed upon an encoder-decoder paradigm and incorporates two complementary modules that jointly enhance representation quality. The SE-convolution Transformer module leverages channel-wise recalibration and global attention to capture long-range dependencies while preserving fine-grained structural cues. In parallel, the deep feature capture module strengthens high-level semantic learning by aggregating multi-scale contextual information and stabilizing feature propagation across layers. At the decoding stage, a dual-decoder refinement strategy further enriches boundary perception by progressively integrating hierarchical features and reinforcing consistency between coarse and detailed predictions. Extensive evaluations on the arterial phase, portal venous phase, and the 3DIRCADb datasets demonstrate that SECT-Net consistently surpasses existing CNNs and Transformer-based models, highlighting its strong adaptability to lesions of varying appearance and contrast. Additional ablation analyses verify the individual contributions of the SECTM and DFCM, confirming their roles in improving localization accuracy and structural completeness. Overall, SECT-Net achieves a favorable balance between segmentation precision, robustness, and computational cost, illustrating its potential for deployment in real-world clinical liver tumor analysis.

## Data Availability

The raw data supporting the conclusions of this article will be made available by the authors, without undue reservation.
